# Supplementary material for: Risk Factors for Neurological Deficits Following Brain Tumor Resection in the Supplementary Motor Area (SMA): A 66-Case Double-Center Study
Source: Cancers (Basel). 2025 Apr 19;17(8):1369. doi: 10.3390/cancers17081369 (PMC12025504; doi:10.3390/cancers17081369)
Supplement: Supplementary file 1 [file cancers-17-01369-s001.zip › cancers-3575840-supplementary.pdf]

Supplementary Materials:

Table S1. Demographic details, clinical history, histopathological diagnosis, and presenting symptoms of the studied population.

Patient Number	*Case Number*	*Gender*	*Age at Surgery*	*History/Comorbidities*	*Pathology*	*WHO Grade*	*Side*	*Presenting Symptoms*
1	1	M	55	family history of cancer	oligodendroglioma	2	lt	balance impairment, concentration and memory difficulties
2	2	M	52	COPD, STEMI, BMI 40.3	oligoastrocytoma	2	lt	3 grand mal seizures, right weakness
3	3	F	67	heart disease	oligoastrocytoma	2	lt	nocturnal seizures
4	4	F	51	left parafalcine meningioma	oligodendroglioma	2	lt	seizures
	5		55		oligodendroglioma	2	lt	-
5	6	M	63	Gilbert's syndrome	astrocytoma	2	rt	left-arm focal seizures evolved into generalized tonic-clonic seizures
6	7	M	51	-	oligodendroglioma	2	rt	2 seizures
	8		53		oligodendroglioma	2	rt	headache
7	9	M	52	primary hypertension, hypercholesterolemia, NSTEMI	oligodendroglioma	2	rt	post-TBI seizures
8	10	M	44	appendicectomy	astrocytoma	2	lt	seizures
	11		48		astrocytoma	2	lt	seizures
9	12	F	37	social anxiety	oligodendroglioma	2	rt	seizures, impaired coordination, cognitive difficulties
10	13	M	56	family history of bowel carcinoma	oligodendroglioma	2	lt	epilepsy
11	14	M	17	-	ganglioglioma	2	rt	epilepsy
12	15	F	10	asthma	ependymoma	2	rt	seizures, dysphasia, memory loss
13	16	M	46	-	oligoastrocytoma	2	rt	seizures

14	17	F	57	meningioma	oligodendroglioma	2	rt	headache, left weakness
15	18	F	69	T2DM, hypertension, retinopathy	astrocytoma	3	rt	sudden weakness, slurring of speech
16	19	M	28	asthma	astrocytoma	2	rt	generalized tonic-clonic seizure
	20		33		astrocytoma	3	rt	2 generalized tonic-clonic seizures
17	21	M	63	asthma, pulmonary embolism	astrocytoma	3	lt	generalized tonic-clonic seizure, right-hand weakness, dysphasia
18	22	F	56	WHO grade II oligodendroglioma	oligodendroglioma	3	rt	left-arm focal seizures
19	23	M	24	asthma	oligoastrocytoma	3	rt	grand mal seizures
	24		32		oligoastrocytoma	3	rt	worsening seizure frequency
20	25	M	37	-	astrocytoma	3	rt	2 generalized tonic-clonic seizures
21	26	M	49	family history of NHL	astrocytoma	3	rt	seizures
22	27	M	29	asthma	astrocytoma	2	lt	generalized tonic-clonic seizure
	28		32		astrocytoma	3	lt	dysphasia, confusion
	29		36		astrocytoma	3	lt	headache
	30		37		astrocytoma	3	lt	-
23	31	M	34	asthma	astrocytoma	3	rt	seizure, left-arm weakness
	32		36		abnormal tissue non-conclusive of tumor recurrence	-	rt	seizures
24	33	F	67	-	astrocytoma	3	lt	headache, left weakness

25	34	F	67	-	glioblastoma	4	rt	focal seizures, weakness, left abnormal sensation
26	35	M	47	-	glioblastoma	4	lt	right-sided face weakness, dysphasia, left-arm paresthesia
27	36	F	53	family history of GBM	glioblastoma	4	lt	right-hand weakness, dysphasia
	37		57		glioblastoma	4	lt	seizures
28	38	M	58	asthma	glioblastoma	4	rt	left-arm weakness, dysphasia, left-arm reduced sensation
29	39	M	62	-	glioblastoma	4	lt	seizures
30	40	M	58	-	glioblastoma	4	lt	seizures
	41		60		glioblastoma	4	lt	-
31	42	F	53	hypercholesterolemia, varicose vein	glioblastoma	4	lt	right-leg clumsiness
32	43	F	52	-	glioblastoma	4	rt	seizures
	44		53		glioblastoma	4	rt	-
33	45	F	47	-	glioblastoma	4	lt	headache
	46		49		glioblastoma	4	lt	dysphasia
34	47	M	34	low back pain	glioblastoma	4	lt	right weakness, dysphasia
35	48	M	59	-	glioblastoma	4	lt	headache
36	49	M	75	-	glioblastoma	4	lt	seizures
37	50	M	70	-	glioblastoma	4	rt	seizures
38	51	F	78	-	glioblastoma	4	rt	headache
39	52	M	59	-	glioblastoma	4	lt	right weakness
40	53	F	62	-	glioblastoma	4	lt	-
41	54	M	64	-	glioblastoma	4	rt	headache
42	55	F	52	-	glioblastoma	4	lt	seizure, right weakness
43	56	F	67	-	glioblastoma	4	rt	seizure

44	57	M	68	-	glioblastoma	4	rt	headache, left weakness
45	58	F	62	glioblastoma	glioblastoma	4	lt	right weakness
46	59	M	59	right lung's adenocarcinoma, right chest wall metastasis	adenocarcinom metastasis	-	rt	generalized seizure
47	60	M	63	right lung's adenocarcinoma	adenocarcinom metastasis	-	rt	left weakness
48	61	M	73	right lung's adenocarcinoma, multiple liver metastases	adenocarcinom metastasis	-	lt	right weakness
49	62	F	69	right lung's adenocarcinoma	adenocarcinom metastasis	-	rt	-
50	63	M	66	right small cell lung cancer	small cell metastasis	-	lt	headache, right weakness
51	64	F	68	right undifferentiated lung cancer	undifferentiated metastasis	-	lt	headache
52	65	M	59	left lung's adenocarcinoma	adenocarcinom metastasis	-	rt	seizures
53	66	M	66	diffuse large B cell lymphoma	large B cell metastasis	-	lt	-

Table S2. Muscles where couples of sub-dermal needle electromyography (EMG) electrodes were placed to record motor-evoked potentials (MEPs).

Name	*Acronym*	*Site*
Frontalis	F	face
Orbicularis Oculi	OOc	
Masseter	Ms	
Orbicularis Oris	OOr	
Mentalis	Mn	
Platysma	P	neck
Trapezius	T	
Deltoid	D	shoulder
Biceps Brachii	BB	arm
Triceps Brachii	TB	
Extensor Digitorum Carpi	EDC	forearm

Flexor Digitorum Superficialis	FDS	
First Dorsal Interosseus	FDI	
Abductor Pollicis Brevis	APB	hand
Abductor Digiti Minimi	ADM	
Vastus Lateralis	VL	tight
Gastrocnemius	G	leg
Tibialis Anterior	TA	
Abductor Hallucis	AH	foot

Table S3. Motor paradigms, fMRI activation of the SMA, minimum distance between the tumor and the area of PMC fMRI activation, verbal paradigms, dominant hemisphere for language, and minimum distance between the CST and the tumor.

Patients						*fMRI*					*Tractography*
						motor paradigms			*language paradigms*		
Pt	*Case*	*Gender*	*Age*	*WHO grade*	*Side*	tasks	SMA activation	M1-tumor distance (mm)	tasks	dominant hemisphere	*CST-tumor distance (mm)*
1	1	M	55	2	lt	both hands	yes	25.93	rhyming<br>word generation<br>verb generation	lt	4.5
2	2	M	52	2	lt	right hand<br>right foot	yes	19.62	-		1.6
3	3	F	67	2	lt	right hand	no	10.34	rhyming<br>word generation<br>verb generation	lt	2.0
4	4	F	51	2	lt	-			rhyming<br>word generation<br>verb generation	lt	-
	5		55	2	lt	tongue	yes	20.10	rhyming<br>word generation<br>verb generation	lt	4.1
5	6	M	63	2	rt	left hand<br>left foot	no	18.72	-		3.9
6	7	M	51	2	rt	-			-		-
	8		53	2	rt	-			-		-
7	9	M	52	2	rt	-			-		-

8	10	M	44	2	lt	-			-		-
	11		48	2	lt	right hand<br>right foot	no	7.12	-		1.8
9	12	F	37	2	rt	both hands<br>left foot	yes	12.49	-		3.5
10	13	M	56	2	lt	right hand<br>right foot	yes	0	-		0
11	14	M	17	2	rt	-			-		-
12	15	F	10	2	rt	left hand<br>tongue	no	0	rhyming<br>word generation<br>verb generation	bilat.	0
13	16	F	69	3	rt	-			-		-
14	17	M	28	2	rt	both hands	yes	21.73	-		4.3
	18		33	3	rt	left hand	yes	12.80	-		2.0
15	19	M	63	3	lt	both hands<br>both feet	yes	8.26	-		0.7
16	20	F	56	3	rt	both hands	yes	21.82	rhyming<br>word generation<br>verb generation	lt	4.2
17	21	M	24	3	rt	-			-		-
	22		32	3	rt	left hand<br>left foot	yes	10	word generation	lt	1.1
18	23	M	37	3	rt	left hand	yes	12.29	-		2.0
19	24	M	49	3	rt	-			-		-
20	25	M	29	2	lt	-			-		-
	26		32	3	lt	-			-		-
	27		36	3	lt	-			-		-
	28		37	3	lt	-			-		-
21	29	M	34	3	rt	left hand	yes	26.99	-		5
	30		36	-	rt	-			-		-
22	31	F	67	4	rt	-			-		-
23	32	M	47	4	lt	right hand<br>tongue	yes	4.98	-		0.7
24	33	F	53	4	lt	-			-		-
	34		57	4	lt	-			-		-

25	35	M	58	4	rt	left hand	yes	13	-		2.0
26	36	M	62	4	lt	right hand	no	16.1	-		4.5
27	37	M	58	4	lt	-			-		-
	38		60	4	lt	right hand	yes	19.24	-		5
28	39	F	53	4	lt	right hand<br>right toes	yes	14.5	-		2.2
29	40	F	52	4	rt	-			-		-
	41		53	4	rt	left hand<br>left foot	no	9.21	-		0
30	42	F	47	4	lt	-			-		-
	43		49	4	lt	-			rhyming<br>word generation<br>verb generation	rt	-
31	44	M	34	4	lt	right hand	yes	11.31	-		2.9
32	45	M	59	metastasis	rt	left hand	no	17.02	-		4.0
33	46	M	63	metastasis	rt	-			-		-
34	47	M	73	metastasis	lt	-			-		-

Table S4. Intraoperative neurophysiology monitoring data. SSEP = somatosensory evoked potential; MEP = motor-evoked potential; sEMG = spontaneous electromyography; EEG = electroencephalography; CS = cortical stimulation; PMC = primary motor cortex; SMA = supplementary motor area; sCS = subcortical stimulation; cMEP = continuous motor-evoked potentials; BL = baseline; mA = milliampere; mm = muscles; * = low amplitude MEP (<50 microvolts); / = no muscles showing MEPs; nr = not reported; - = not performed. Muscles' acronyms (see Table S2): F, OOc, Ms, OOr, Mn, P, T, D, BB, TB, EDC, FDS, FDI, APB, ADM, VL, G, TA, AH.

Patients		***Intraoperative Neurophysiology Monitoring***								
		SSEP		MEP					sEMG	EEG
				CS (mA: mm showing MEPs)			cMEP			
Pt	Case	nerve stimulated	phase reversal	PMC (M1)	SMA	sCS (mA: mm showing MEPs)	BL (mA: mm set as BL)	mm showing >50% voltage decrease from BL	discharges	wave anomalies
1	1	-		20: EDC, FDI, APB	25: /	25: /	-		none	-
2	2	median<br>posterior tibial	yes<br>no	15.5: EDC, FDI, APB	20.1: TA	15.3: TA, AH	12: EDC, FDI, APB	EDC, FDI, APB	none	-

3	3	-		15: EDC, ADM	18.2: /	15.8: FDI, ADM. TA	-		-	-
4	4	median	yes	nr		-	-		-	poly spike activity
	5	-		8: OOc EDC, FDI APB	12: /	-	-		none	-
5	6	median	no	nr		-	-		-	-
6	7	-		-		-	-		-	-
	8	-		12: TA, AH 14: EDC, FDI, APB, ADM	14: /	-	-		none	-
7	9	-		nr		-	-		-	-
8	10	median	yes	nr		-	-		-	none
	11	median	no	20: D, FDS, APB	25: D	-	-		-	poly spike activity
9	12	-		10: G*, TA* 12: EDC, VL, TA, G, AH	12: /	12: TA, AH	12: VL, TA, G, AH	VL, TA, G, AH	none	-
10	13	posterior tibial	yes	nr		-	-		-	none
11	14	-		-		-	-		-	-
12	15	-		18: /	18: /	-	-		none	-
13	16	-		12: D, BB, APB	20: APB	12: APB	12: BB	BB	-	-
14	17	-		-		-	-		-	-
15	18	-		12: APB, FDI*	12: /	14: APB, ADM	13: APB	none	none	-
16	19	median	yes	nr		-	-		-	-
	20	-		-		-	-		-	-
17	21	median	yes	nr		-	-		-	-
18	22	median	yes	nr		-	-		none	-
19	23	-		-		-	-		-	-
	24	median	yes	5: EDC 10: FDI, APB 15: D, TA, AH	20: D, EDC, FDI, TA, AH	-	10: EDC, FDI, APB	none	none	-
20	25	posterior tibial	yes	nr		-	-		-	-

21	26	ulnar	yes	nr		-	-		-	-
	27	-		-		-	-		-	-
	28	-		-		-	-		-	-
22	29	-		12: FDI, APB	12: /	8: EDC, FDI, APB 12: D	-		none	-
	30	-		-		12: EDC, FDI, APB ADM	-		-	-
23	31	median	no	nr		-	-		-	-
	32	-		nr		-	-		-	-
24	33	-		-		-	-		-	-
25	34	-		15.2: EDC, FDI, APB, TA	17: /	-	-		none	-
26	35	-		-		-	-		-	-
27	36	median	yes	20.7: APB	20.7: /	-	20: APB	none	none	-
	37	-		-		-	-		-	-
28	38	median	yes	15: EDC, FDI, APB	18: /	15: EDC, FDI, APB	-		none	-
29	39	median	yes	nr		-	-		-	-
30	40	-		-		-	-		-	-
	41	-		15: EDC, FDI, APB	18: /	15: EDC*	-		-	-
31	42	median	yes	nr		-	-		-	-
32	43	median	no	15: EDC, FDI, APB	15: /		15: EDC, FDI, APB	none	none	-
	44	-		15: EDC, FDI, APB, TA, AH	19: /	-	-		none	-
33	45	median	yes	nr		-	-		-	-
	46	-		14: /	14: /	-	-		-	none
34	47	-		25: EDC, FDI, APB	25: /	25: APB, VL, BF, TA, AH	-		none	-
35	48	-		-		-	-		-	-
36	49	-		15: G, AH	21: G	8: G	15: G, AH	G, AH	-	-
37	50	-		-		-	-		-	-
38	51	-		-		-	-		-	-
39	52	-		-		-	-		-	-
40	53	-		-		-	-		-	-
41	54	-		15: G, AH	25: AH	15: AH	-		-	-

42	55	-		-		-	-		-	-
43	56	-		12: D, BB	21: BB	15: BB	-		-	-
44	57	-		-		-	-		-	-
45	58	-		-		-	12: D, BB	D, BB	-	-
46	59	posterior tibial	yes	nr		nr	-		-	-
47	60	median	yes	nr		-	-		-	-
48	61	median	yes	nr		-	-		-	-
49	62	-		12: BB	18: /	-	-		-	-
50	63	-		15: BB	19:/	-	-		-	-
51	64	-		-		-	-		-	-
52	65	-		-		-	-		-	-
53	66	-		-		-	-		-	-

Table S5. Postoperative outcomes. pSMA = partial SMA syndrome; cSMA = complete SMA syndrome; pld = permanent language deficit.

Patients		*Postoperative Outcomes*							
		motor deficits			*language deficits*				
Pt	*Case*	*nature of deficit*	*grade*	*recover*	*nature of deficit*	*grade*	*recover*	*others*	*syndrome*
1	1	-	A	-	-	A	-	-	-
2	2	right-leg weakness	B	yes	-	A	-	seizures	pSMA
3	3	left weakness	B	yes	dysphasia	B	yes	-	pSMA
4	4	-	A	-	dysphasia	B	yes	-	pSMA
	5	-	A	-	dysphasia	B	yes	-	pSMA
5	6	-	A	-	-	A	-	seizures, left-arm tremor	-
6	7	-	A	-	-	A	-	headache	-
	8	-	A	-	-	A	-	headache	-
7	9	-	A	-	-	A	-	-	-
8	10	right bradykinesia	B	yes	-	A	-	-	pSMA
	11	right weakness	B	yes	dysphasia	B	yes	-	pSMA
9	12	left-leg weakness	B	yes	-	A	-	sensory disturbances and blurring of vision	pSMA
10	13	-	A	-	dysphasia	B	yes	-	pSMA
11	14	-	A	-	-	A	-	-	-
12	15	-	A	-	dysphasia	B	no	dizziness and tingling	pld
13	16	-	A	-	-	A	-	-	-
14	17	-	A	-	-	A	-	-	-
15	18	left-arm weakness	B	yes	-	A	-	-	pSMA
16	19	left-arm weakness	B	yes	dysphasia	B	yes	-	pSMA
	20	left weakness	B	yes	-	A	-	-	pSMA
17	21	right weakness	B	yes	dysphasia	B	yes	-	pSMA
18	22	left weakness	B	yes	-	A	-	-	pSMA

19	23	-	A	-	-	A	-	-	-
	24	right paralysis	C	yes	aphasia	C	yes	-	cSMA
20	25	left-hand weakness	B	yes	-	A	-	short-term memory loss	pSMA
21	26	-	A	-	-	A	-	-	-
22	27	-	A	-	-	A	-	-	-
	28	-	A	-	-	A	-	-	-
	29	right-arm weakness	B	yes	-	A	-	visual disturbances	pSMA
	30	-	A	-	-	A	-	-	-
23	31	-	A	-	-	A	-	-	-
	32	-	A	-	-	A	-	-	-
24	33	-	A	-	-	A	-	-	-
25	34	left weakness	B	yes	-	A	-	-	pSMA
26	35	right weakness	B	yes	dysphasia	B	yes	-	pSMA
27	36	-	A	-	-	A	-	-	-
	37	right-arm weakness	B	yes	dysphasia	B	yes	-	pSMA
28	38	-	A	-	-	A	-	-	-
29	39	-	A	-	-	A	-	-	-
30	40	right weakness	B	yes	-	A	-	-	pSMA
	41	right weakness	B	yes	-	A	-	-	pSMA
31	42	right weakness	B	yes	dysphasia	B	yes	-	pSMA
32	43	-	A	-	-	A	-	-	-
	44	-	A	-	-	A	-	-	-
33	45	-	A	-	-	A	-	short-term memory loss	-
	46	-	A	-	-	A	-	-	-
34	47	-	A	-	dysphasia	B	yes	-	pSMA
35	48	-	A	-	-	A	-	-	-
36	49	left-leg weakness	B	yes	-	A	-	-	pSMA
37	50	left-leg weakness	B	yes	-	A	-	-	pSMA
38	51	-	A	-	-	A	-	-	-
39	52	-	A	-	-	A	-	-	-
40	53	-	A	-	-	A	-	-	-
41	54	-	A	-	-	A	-	-	-
42	55	-	A	-	-	A	-	-	-
43	56	-	A	-	-	A	-	-	-
44	57	-	A	-	-	A	-	-	-
45	58	right-arm weakness	B	yes	-	A	-	-	pSMA
46	59	-	A	-	-	A	-	-	-
47	60	-	A	-	-	A	-	-	-

48	61		right-foot weakness	B	yes	-	A	-	-	pSMA
49	62		-	A	-	-	A	-	-	-
50	63		-	A	-	-	A	-	-	-
51	64		-	A	-	-	A	-	-	-
52	65		-	A	-	-	A	-	-	-
53	66		-	A	-	-	A	-	-	-

Table S6. Analysis of clinical risk factors in the entire patient cohort. * = the previous treatment for SMA tumor was not considered as part of the clinical history, because regarded as an independent variable; RT = radiotherapy; CHT = chemotherapy; ** = metastases were regarded as high-grade tumors; *** = motor and language deficits were analyzed separately.

Clinical Factors	***Immediate post-op deficits***							
	motor deficits				language deficits			
	p Value	yes	none		*p* Value	yes	none	
Age	0.16406694			Tot.	0.13982242			Tot.
< 50 years		9	13	22		6	16	22
> 50 years		14	30	44		7	37	44
	Tot.	23	43	66	Tot.	13	53	66
Past medical history*	0.20285078			Tot.	0.00516368			Tot.
any comorbidity/family-history		14	25	39		12	27	39
none		9	18	27		1	26	27
	Tot.	23	43	66	Tot.	13	53	66
Previous treatment	0.05887430			Tot.	0.20307985			Tot.
resection and/or RT-CT		8	7	15		4	11	15
none		15	36	51		9	42	51
	Tot.	23	43	66	Tot.	13	53	66
Tumor grade**	0.20514422			Tot.	0.05198844			Tot.
high		18	31	49		7	42	49
low		5	12	17		6	11	17
	Tot.	23	43	66	Tot.	13	53	66
Pre-op deficits***	0.14073190			Tot.	0.05543355			Tot.

yes		9	12	21		4	5	9
none		14	31	45		9	48	57
	Tot.	23	43	66	Tot.	13	53	66

Table S7. Analysis of clinical risk factors in the patients with gliomas. * = the previous treatment for SMA tumor was not considered as part of the clinical history, because regarded as an independent variable; RT = radiotherapy; CHT = chemotherapy; *** = motor and language deficits were analyzed separately.

Clinical Factors	***Immediate post-op deficits***							
	motor deficits				language deficits			
	p Value	yes	none		*p* Value	yes	none	
Age	0.20446256			Tot.	0.19737897			Tot.
< 50 years		9	13	22		6	16	22
> 50 years		13	23	36		7	29	36
	Tot.	22	36	58	Tot.	13	45	58
Past medical history*	0.17195283			Tot.	0.00120746			Tot.
any comorbidity/family-history		13	18	31		12	19	31
none		9	18	27		1	26	27
	Tot.	22	36	58	Tot.	13	45	58
Previous treatment	0.08971771			Tot.	0.24393396			Tot.
resection and/or RT-CT		8	7	15		4	11	15
none		14	29	43		9	34	43
	Tot.	22	36	58	Tot.	13	45	58
Tumor grade	0.16685345			Tot.	0.08817281			Tot.
high		17	24	41		7	34	41
low		5	12	17		6	11	17
	Tot.	22	36	58	Tot.	13	45	58
Pre-op deficits***	0.18063642			Tot.	0.08203287			Tot.
yes		8	10	18		4	5	9
none		14	26	40		9	40	49
	Tot.	22	36	58	Tot.	13	45	58

Table S8. Analysis of clinical risk factors in the patients with metastases. * = the previous treatment for SMA tumor was not considered as part of the clinical history, because regarded as an independent variable; RT = radiotherapy; CHT = chemotherapy; *** = motor and language deficits were analyzed separately.

Clinical Factors	***Immediate post-op deficits***							
	motor deficits				language deficits			
	p Value	yes	none		*p* Value	yes	none	
Age	1			Tot.	1			Tot.
< 50 years		0	0	0		0	0	0
> 50 years		1	7	8		0	8	8
	Tot.	1	7	8	Tot.	0	8	8
Past medical history*	1			Tot.	1			Tot.
any comorbidity/family-history		1	7	8		0	8	8
none		0	0	0		0	0	0
	Tot.	1	7	8	Tot.	0	8	8
Previous treatment	1			Tot.	1			Tot.
resection and/or RT-CT		0	0	0		0	0	0
none		1	7	8		0	8	8
	Tot.	1	7	8	Tot.	0	8	8
Pre-op deficits***	0.375			Tot.	1			Tot.
yes		1	2	3		0	0	0
none		0	5	5		0	8	8
	Tot.	1	7	8	Tot.	0	8	8

Table S9. Analysis of preoperative risk factors in the entire patient cohort. SMA = supplementary motor area; PMC = primary motor cortex; CST = corticospinal tract.

Pre-op Factors	***Immediate post-op deficits***							
	motor deficits				language deficits			
	p Value	yes	none		*p* Value	yes	none	
SMA motor activation	0.0446224<br>3			Tot.				
yes		12	6	18				
no		1	5	6				
	Tot.	13	11	24				

Dominant hemisphere for language					0.26785714			Tot.
homolateral						4	1	5
contralateral						1	2	3
					Tot.	5	3	8
PMC-tumour distance	0.34086575			Tot.				
> 10 mm		9	8	17				
< or = 10 mm		4	3	7				
	Tot.	13	11	24				
CST-tumor distance	0.00548905			Tot.				
> 2 mm		7	23	30				
< or = 2 mm		9	4	13				
	Tot.	16	27	43				

Table S10. Analysis of preoperative risk factors in the patients with gliomas. SMA = supplementary motor area; PMC = primary motor cortex; CST = corticospinal tract.

Pre-op Factors	***Immediate post-op deficits***							
	motor deficits				language deficits			
	p Value	yes	none		*p* Value	yes	none	
SMA motor activation	0.08113168			Tot.				
yes		12	6	18				
no		1	4	5				
	Tot.	13	10	23				
Dominant hemisphere for language					0.26785714			Tot.
homolateral						4	1	5
contralateral						1	2	3
					Tot.	5	3	8
PMC-tumour distance	0.31873161			Tot.				
> 10 mm		9	8	17				
< or = 10 mm		4	2	6				
	Tot.	13	10	23				

CST-tumor distance	0.0192193 8			Tot.
> 2 mm		7	17	24
< or = 2 mm		9	4	13
	Tot.	16	21	37

Table S11. Analysis of preoperative risk factors in the patients with metastases. SMA = supplementary motor area; PMC = primary motor cortex; CST = corticospinal tract.

Pre-op Factors	*Immediate post-op deficits*			
	motor deficits			
	p Value	yes	none	
SMA motor activation	1			Tot.
yes		0	0	0
no		0	1	1
	Tot.	0	1	1
PMC-tumour distance	1			Tot.
> 10 mm		0	0	0
< or = 10 mm		0	1	1
	Tot.	0	1	1
CST-tumor distance	1			Tot.
> 2 mm		0	6	6
< or = 2 mm		0	0	0
	Tot.	0	6	6

Table S12. Analysis of intraoperative risk factors in the entire patient cohort. MEPs = motor-evoked potentials; GTR = gross total resection; STR = subtotal resection.

Intra-op Factors	*Immediate post-op deficits*							
	motor deficits				language deficits			
	p Value	yes	none		*p* Value	yes	none	
MEPs monitoring	0.1001839 2			Tot.				
yes		18	27	45				
no		5	16	21				
	Tot.	23	43	66				
Language mapping					0.0440988 2			Tot.
yes						3	2	5
no						10	51	61

					Tot.	13	53	66
Extent of resection	0.07351605			Tot.	0.04035376			Tot.
GTR		8	23	31		3	28	31
STR		15	20	35		10	25	35
	Tot.	23	43	66	Tot.	13	53	66

Table S13. Analysis of intraoperative risk factors in the patients with gliomas. MEPs = motor-evoked potentials; GTR = gross total resection; STR = subtotal resection.

Intra-op Factors	*Immediate post-op deficits*							
	motor deficits				language deficits			
	p Value	yes	none		*p* Value	yes	none	
MEPs monitoring	0.1352358			Tot.				
yes		17	23	40				
no		5	13	18				
	Tot.	22	36	58				
Language mapping					0.049693199			Tot.
yes						3	2	5
no						9	44	53
					Tot.	12	46	58
Extent of resection	0.1416390			Tot.	0.103029855			Tot.
GTR		7	16	23		3	20	23
STR		15	20	35		10	25	35
	Tot.	22	36	58	Tot.	13	45	58

Table S14. Analysis of intraoperative risk factors in the patients with metastases. MEPs = motor-evoked potentials; GTR = gross total resection; STR = subtotal resection.

Intra-op Factors	*Immediate post-op deficits*							
	motor deficits				language deficits			
	p Value	yes	none		*p* Value	yes	none	
MEPs monitoring	0.625			Tot.				
yes		1	4	5				
no		0	3	3				
	Tot.	1	7	8				

Language mapping					1			Tot.
yes						0	0	0
no						1	7	8
					Tot.	1	7	8
Extent of resection	1			Tot.	1			Tot.
GTR		1	7	8		0	8	8
STR		0	0	0		0	0	0
	Tot.	1	7	8	Tot.	0	8	8
